# Vertebrobasilar circulation hemorrhages in childhood primary angiitis of the central nervous system

**DOI:** 10.4322/acr.2021.391

**Published:** 2022-07-18

**Authors:** Ahmad Hassan, Kieren Allinson

**Affiliations:** 1 Addenbrooke’s Hospital, Department of Acute Medicine, Cambridge, United Kingdom; 2 Addenbrooke’s Hospital, Department of Neuropathology, Cambridge, United Kingdom

**Keywords:** Autopsy, Brain Death, Vasculitis, Central Nervous System, Diagnosis, Hemorrhagic Stroke, Mortality

## Abstract

Childhood primary angiitis of the CNS (cPACNS) is a poorly understood, rare, and diagnostically challenging neurologic disease. We describe an unusual and autopsy-confirmed case of cPACNS presenting as vertebrobasilar circulation hemorrhagic strokes in a 4-year-old girl. The presentation and clinical features were inconsistent with primary CNS vasculitis and skewed the diagnosis. Autopsy and histopathological analyses revealed a progressive lymphocytic vasculitis affecting the medium to large vessels of vertebrobasilar circulation and sparing the anterior circulation. It is imperative to raise the index of suspicion for cPACNS in any case of unusual or unexplained neurological presentation, especially in the absence of cerebrovascular risk factors and/or coagulation disorders.

## INTRODUCTION

Childhood Primary Angiitis of the Central Nervous System (cPACNS) is an increasingly recognized but diagnostically challenging autoinflammatory central nervous system disease of unknown etiology.^1^ It causes immune-mediated destruction of the small, medium, or large-sized blood vessels within the brain and spinal cord. The resulting clinical presentation varies in terms of; i) affected anatomical territory, which can be focal or diffuse; ii) the type of lesion, which in a majority of cases is an ischemic or a mass lesion;^2^ iii) pathological progression, which can be benign or progressive, and iv) disease pattern, which can be constant or relapsing/remitting.^3^

It is diagnostically challenging because; i) It is a relatively rare disease with no ethnic, gender, or age group preponderance,^4^ ^,^ ^5^ ii) There is no distinct clinical picture, and the cases present with a variety of neurological syndromes,^6^ ^-^ ^8^ iii) There is no universal diagnostic criterion so far,^9^ iv) There is no specific diagnostic test and confirmation relies on histology, v) Brain and spinal cord are relatively unsafe to biopsy, and vi) It is a poorly understood disease with diverse immune effectors and no precise immunopathological description (for example granulomatous or lymphocytic vasculitis).^6^ Consequently, there are very limited therapeutic options, and it is at best only controllable with immunosuppression and antiplatelet therapy.^2^ ^,^ ^10^

Herein, we report an autopsy confirmed cPACNS case, presenting as a posterior cranial fossa hemorrhagic stroke, resulting from aneurysms of vertebrobasilar circulation arteries.

## CASE REPORT

**Figure 1:**
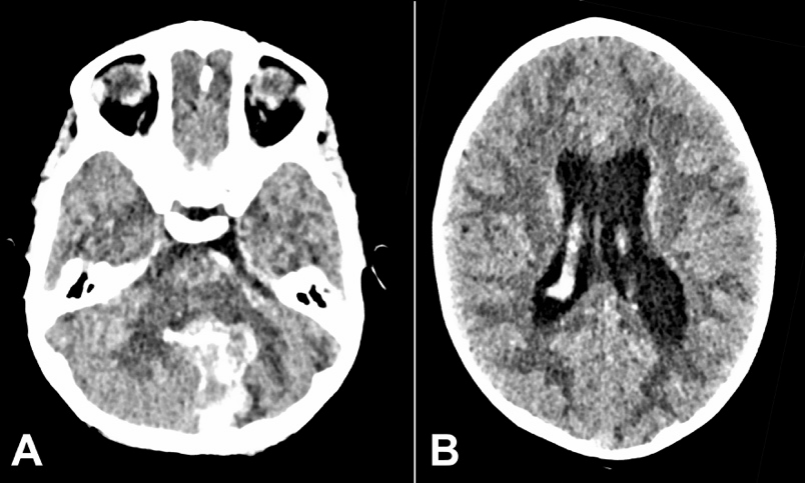
**A –** Brain CT on the first presentation. Intracerebral hemorrhage in the cerebellar vermis and medial aspect of the left cerebellar hemisphere with intraventricular extension; **B –** Brain CT on first presentation showing supra-tentorial hydrocephalus.

A 4-years-old, previously healthy girl, presented to the emergency department after an episode of drowsiness and unwitnessed fall. Symptoms included headache, vomiting, and fluctuating consciousness levels. Initial GCS with ambulance crew ranged from 10/15-13/15, which deteriorated to 7/15 at hospital presentation. She was intubated/ventilated and an urgent CT head revealed a large hemorrhage into the cerebellar vermis and left cerebellar hemisphere, extending into the ventricular system and causing supratentorial hydrocephalus. (Figures 1A and 1B).

**Figure 2:**
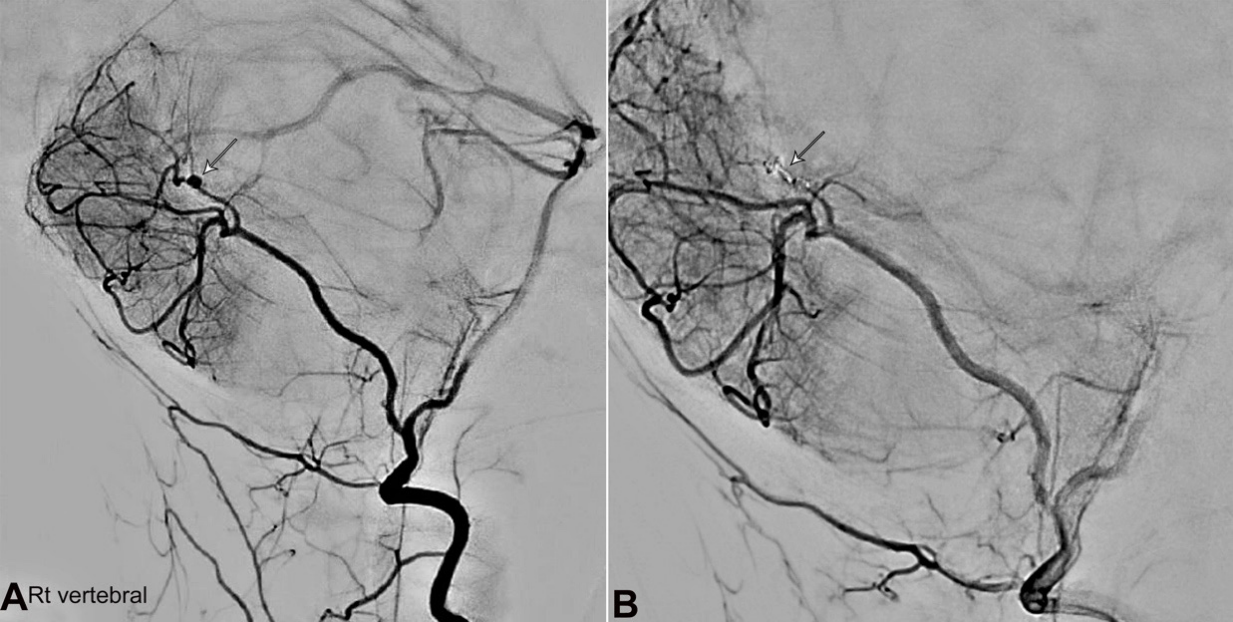
**A –** Digital Subtraction Angiogram (DSA) on the first presentation before coiling of the aneurysm. Arrows showing aberrant right PICA and irregular distal branches and a distal aneurysm; **B –** Digital Subtraction Angiography (DSA) after coiling of the aneurysm.

A CT angiogram (CTA) revealed a ruptured aneurysm of the posterior inferior cerebellar artery (PICA) branch. An external ventricular drain (EVD) was inserted for intracranial pressure (ICP) control, and she was admitted to PICU. The aneurysm was coiled, the hematoma evacuated, and a digital subtraction angiography (DSA) revealed an aberrant branch of PICA with a distal aneurysm (Figures 2A and 2B).

Histological examination of tissue from the roof of the 4th ventricle showed normal choroid plexus. The patient stayed in PICU over the next 4 weeks, during which she developed EVD-associated meningitis and was treated with antibiotics. The EVD was replaced with a ventriculoperitoneal (VP) shunt. She clinically recovered and was discharged with ongoing physiotherapy and rehabilitation. This episode of brain hemorrhage was understood to be secondary to a fall and traumatic PICA aneurysm rupture.

During this hospital admission, all blood tests (including liver function, kidney function, and hematology) remained unremarkable except for an elevated CRP, 36 (0-6) mg/L, and a positive lupus anticoagulant (which was deemed insignificant due to concurrent low molecular weight heparin and a phospholipid correction of 22.14%). Lupus anticoagulant was not repeated. CSF examination on multiple occasions revealed high RBC counts (range 47,160 x 10^6^/L to 82 x 10^6^/L), a few polymorphonuclear cells (2x10^6^/L to 8x10^6^/L), a few lymphocytes (4x10^6^/L to 10x10^6^/L), and no organisms. WBC: RBC ranged from 0.1 to 17. The highest WBC: RBC was 17 (lymphocyte count was 10x10^6^/L), which corresponded to the episode of EVD-associated meningitis.

Over the course of the next year, she underwent genetic screening for vasculopathies; i) Thoracic aorta aneurysm/dissection screen including TGF-β signaling cascade proteins and smooth muscle contractile elements proteins, ii) Hereditary hemorrhagic telangiectasia, iii) Fabry’s disease, iv) Collagenopathies & Ehlers-Danlos syndrome, v) Next-generation sequencing/whole-genome sequencing. None of these tests identified any mutations or polymorphisms that could explain a vasculopathy. Echocardiogram, renal ultrasound, and retinal exam were normal. A surveillance MRI and MRA at 10 months did not identify any brain or vascular lesions. Clinical progress remained well, with only one hospital presentation due to headache and vomiting, which resolved with simple analgesia and antiemetics.

**Figure 3:**
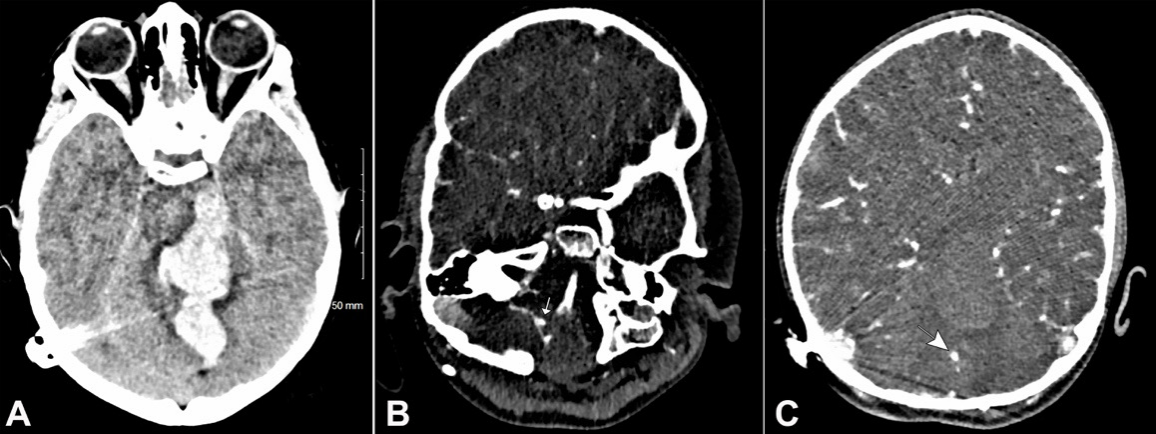
CT brain on the second presentation. **A –** Intracerebral hemorrhage in the cerebellar vermis with extension into the distorted fourth ventricle, the third and lateral ventricles, the quadrilateral cistern, and the left cerebral peduncle with adjacent vasogenic edema; **B –** CTA showing aneurysm of the right superior cerebellar artery (arrow); **C –** Proximal aneurysm of right PICA (arrow).

18 months after the first hospital admission, she experienced sudden onset severe headache, one grand mal seizure lasting 3 minutes, and vomiting, at rest, followed by loss of conscious level - best GCS 04/15 (E2V1M1). She was intubated on scene and an immediate CT head depicted a large intracranial hemorrhage centered onto the cerebellar vermis and extending into the brainstem, ventricular system, the quadrilateral cistern, and the left cerebral peduncle, with adjacent vasogenic edema. CT angiogram revealed another proximal right PICA aneurysm and a further superior cerebellar artery aneurysm (Figures 33B, and 3C).

Clinically, her GCS remained 3-4/15 and she developed decerebrate posturing, Cushing's response, and fixed pupils. The location and extent of the bleed deemed this not survivable and life-sustaining treatment was withdrawn. She passed away after one day.

## AUTOPSY PRESENTATION

A neuropathological autopsy was conducted at the coroner’s request. There were no external features of any dysmorphism. Macroscopic examination of the central nervous system revealed extensive hemorrhage centered on the cerebellar vermis, mesencephalon, 4^th^ ventricle, upper pons, left midbrain, and left cerebral crus with extension into the 3^rd^ and lateral ventricles. The circle of Willis and intracranial arteries appeared normal.

**Figure 4:**
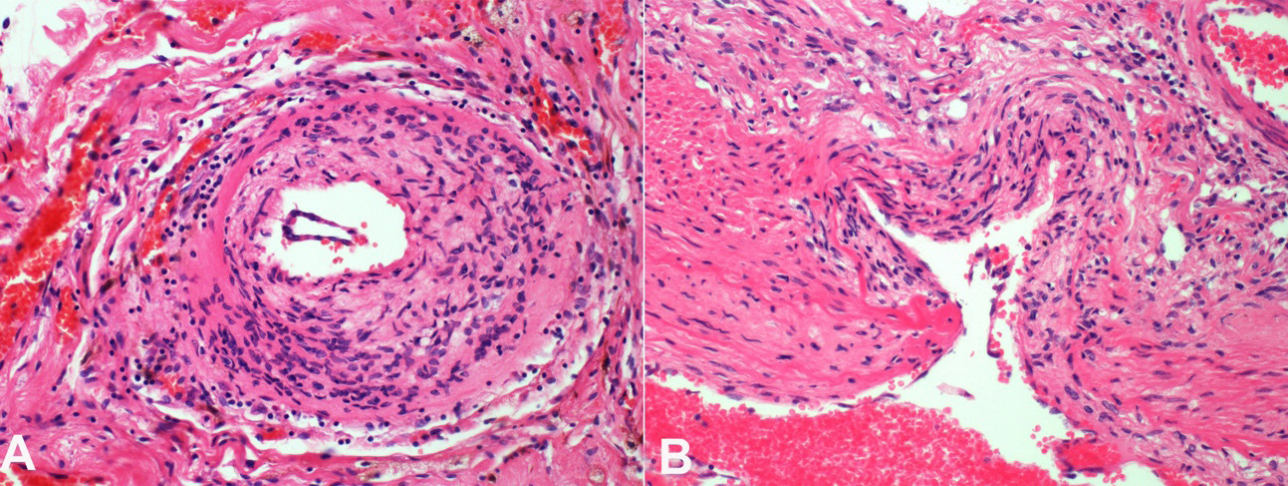
Photomicrograph of the vertebrobasilar circulation vessel. **A –** vertebrobasilar circulation artery showing diffuse lymphohistiocytic infiltrate; **B –** Histopathology of vertebrobasilar circulation artery showing loss of internal elastic lamina and foci of fibrinoid necrosis.

Histological examination showed a multifocal vasculitis involving medium to large-sized arteries of the entire vertebrobasilar system (Figures 4A and 4B). Vasculitis foci ranged from acute active lesions to old healed lesions. Acute lesions had transmural lymphohistiocytic infiltrates, loss of internal elastic lamina, and foci of fibrinoid necrosis. Older lesions comprised of intimal hyperplasia adjacent to thin and scarred smooth muscle media. Several medium and large-sized arteries demonstrated severe stenosis secondary to intimal hyperplasia and fibrosis. There were no granulomas. Sampled anterior circulation vessels did not show any vasculitis. Leptomeninges were normal. No other pathology was identified.

Neuropathologists' impression implied cPACNS. Moyamoya disease was also considered, but histopathological features were not consistent. A systemic vasculitis could not be completely excluded at autopsy since other systems were not examined, although the patient did not have any clinical manifestations of an underlying autoimmune disease.

## DISCUSSION

cPACNS is an immune-mediated vasculitis confined to the central nervous system. The exact morbidity and mortality are unknown. It is clinicopathologically similar to the adult primary angiitis of CNS (PACNS), first identified by Harbitz in 1922 and described by Calabrese in 1988.^1^ ^,^ ^11^ The exact etiology is unascertained, but *Varicella Zoster* or *Mycoplasma* infections are considered possible triggers.^5^ Histological analysis has provided some explanation to the pathophysiology. Immune cell infiltration (predominantly T-cells), brought on by a poorly understood interplay between chemokines/cytokines (IL-6, IL-8, CXCL1, CXCL10), matrix metalloproteinases (MMPs), and tissue inhibitors of MMPs (TIMPs), destroys blood vessel walls. Granulomatous, lymphocytic, or necrotizing patterns exist with resulting damage to tunica intima or media.^12^ This can produce fibrotic thickening and stenosis that causes an ischemic lesion.^5^ ^,^ ^13^ Concurrent weakening of the vessel wall can cause aneurysms and bleeding.^13^ Lymphocytic patterns are more common in cPACNS compared to PACNS.^12^ cPACNS predominantly involves anterior cerebral circulation and resulting lesions show a preponderance for proximal vessel regions.^14^

Clinical presentation is non-specific and depends on the type of lesion, affected type of vessel (small or medium-large), and vascular territory. The disease may manifest as a non-progressive benign lesion or follow a progressive course.^9^ Headache is a frequently reported symptom and is common to all types of clinical presentations. Cognitive and behavioral abnormalities are common in small vessel vasculitis, while focal neurological deficits (hemiparesis, paresthesia, speech disturbance) and ischemic stroke are prevalent in medium-large vessel disease. Seizures and decreased conscious levels are also reported and are frequent in small-vessel disease or in lesions that cause mass effects.^13^ Hemorrhagic strokes are rarely reported.^15^ ^,^ ^16^ Constitutional symptoms such as fever, fatigue, and aches & pains are also rare.^13^ ^,^ ^15^

The disease can only be confirmed histopathologically,^17^ but as brain or spinal cord biopsy is often risky; imaging modalities are preferred to aid the diagnosis. A combination of investigation tests can increase the diagnostic yield, and for better diagnostic sensitivity, a biopsy of the lesion site guided by imaging is ideal.^12^ Imaging studies such as DSA, MRA, or conventional angiograms provide decent visualization of the vascular structures, but their sensitivity in identifying cPACNS-associated vascular lesions is low.^13^ Nevertheless, DSA is the preferred imaging modality with the best sensitivity, followed by the MRA.^18^ Imaging findings include areas of vascular stenosis and dilatation, delayed contrast enhancement, microbleeds, and increased vessel wall thickness.^19^ CT scans can identify gross abnormalities like hemorrhages and infarcts. MRI can detect T2 or FLAIR sequences that are non-specific but aid in diagnosis when correlated with clinical findings.^6^ ^,^ ^20^ CSF examination can depict an elevated protein level, lymphocytosis, or oligoclonal bands.^9^ ^,^ ^21^ Blood tests can show elevated inflammatory markers such as CRP or ESR. Blood tests can also identify systemic vasculitis, which may be causing the CNS vasculitis originally.^13^ ^,^ ^17^

cPACNS is difficult to diagnose, and differential diagnoses are extensive ranging from genetic vasculopathies to reversible cerebral vasoconstriction syndromes (RCVS) (Table 1).^4^

**Table 1:** Secondary Causes and Mimics of CNS Vasculitis in Children

Primary Systemic Inflammatory Disorders	Takayasu arteritis
	Polyarteritis nodosa
	Kawasaki disease
	Granulomatosis with polyangiitis
	Henoch-Schönlein purpura
	Bechet’s disease
	Microscopic polyangiitis
	Systemic lupus erythematosus
	Juvenile dermatomyositis
	Juvenile Rheumatoid Arthritis
	Inflammatory bowel disease
	Hemophagocytic lymphohistiocytosis
	ADA 2-deficiency
	TREX1 - Associated diseases (e.g., Aicardi-Goutières syndrome)
Systemic Inflammation due to secondary causes	Malignancy
	- E.g., Hodgkin's and non-Hodgkin's lymphoma
	Infections
	- E.g., Varicella zoster virus–post-varicella angiopathy
	Drugs
	- E.g., cocaine (alone or contaminated with levamisole), anti-thyroid drugs, hydralazine, minocycline^*^, anticancer agents, sympathomimetic agents^+^.
Diseases that mimic cPACNS^^^	Thromboembolic diseases
	- E.g., Coagulation disorders, hemoglobinopathies, congenital heart disease
	Reversible vasoconstriction syndrome (RVCS)
	Moyamoya syndrome
	- Primary Moyamoya MYMY1–MYMY6 (e.g., RNF213)
	- Secondary Moyamoya syndrome, e.g., in Down syndrome, sickle cell anemia, neurofibromatosis type 1
	Neurometabolic diseases
	- E.g., Fabry’s disease (GLA), homocystinuria
	Fibromuscular dysplasia
	Migrainous infarction
	Genetic vasculopathies
	- E.g., NOTCH3, HTRA1
	Genetic structural alterations of vessels
	- E.g., COL4A1, ACTA2, MOPD2

* Mimics medium-vessel vasculitis, with ANCA positivity;

^+^ Can cause large vessel vasculitis;

^ These diseases do not have inflammatory infiltrate but mimic the clinical presentation of cPACNS. Here the vascular pathology is secondary to change in vascular tone/vasoconstriction (RVCS, Moyamoya) or due to primary pathology of vascular structure elements or infiltration of metabolic substrates.

Although there are no universal diagnostic criteria; the following criteria were developed by Calabrese and modified by Benseler for cPACNS;^3^ ^,^ ^7^ i) Newly acquired neurological deficit, ii) Angiographic and/or histological features of angiitis within the CNS, iii) No evidence of an underlying systemic disorder that explains the symptoms, iv) Recently developed psychiatric deficits.

Considering the low diagnostic yield of imaging and angiography, a new diagnostic criterion was suggested by Rice and Scolding^17^ that classifies the cPACNS cases into ‘definitive’ disease (with clinical features consistent with CNS vasculitis and histological evidence of CNS angiitis on biopsy or autopsy) and ‘probable’ disease (with clinical features consistent with CNS vasculitis, plus, laboratory and imaging clues of CNS inflammation and without histological evidence of vasculitis).

Our clinical case fell into the category of progressive medium to large vessel vasculitis, which could not be diagnosed before autopsy. Several factors led to the diagnostic uncertainty: i) The initial presentation was masked by a fall and interpreted as traumatic brain injury, ii) No clinical symptoms were reported either before the first or between the first and second episode of brain hemorrhage, iii) The disease involved vertebrobasilar circulation which is unusual in cPACNS, iv) The large intracranial bleed obscured diagnostic clues from CSF analysis, v) DSA and MRI + MRA did not identify any lesions consistent with vasculitis, vi) Vasculitis screen and biopsy were not considered.

## CONCLUSION

cPACNS is a rare and perilous disease with no ethnic, gender, or age group preponderance, no distinct clinical picture, no universal diagnostic criteria, or specific diagnostic test.^4^ ^,^ ^5^ Therapeutic options are limited; it is at best controllable with immunosuppression and antiplatelet therapy with an early diagnosis being crucial for therapeutic success.^2^ ^,^ ^10^ It is imperative to raise the clinical index of suspicion for cPACNS in cases of unusual or unexplained neurological presentation and especially in the absence of cerebrovascular risk factors, systemic inflammation, or coagulation disorders. Advanced diagnostic modalities must be considered, such as high-resolution contrast vessel wall MRI as its sensitivity in identifying vessel wall thickness and narrowing is better than conventional MRI, and MRA.^22^ Elevated CSF cytokine and interleukin levels can provide diagnostic cues.^20^ Elevated Von-Willebrand factor levels have been reported in cPACNS cases and could be included in the diagnostic screen.^23^ A biopsy must be considered in all suspected cases as it is the only reliable investigation if targeted correctly.^12^
